# Etiologies of Dysphonia in Patients Referred to ENT Clinics Based on Videolaryngoscopy

**Published:** 2014-07

**Authors:** Keyvan Kiakojoury, Mehdi Dehghan, Fateme Hajizade, Soraya Khafri

**Affiliations:** 1 *Department of Otorhinolaryngology, Babol University of Medicine Sciences, Mazandaran, Iran.*; 2 *Department of Speech Therapy, Babol University of Medicine Sciences, Mazandaran, Iran.*; 3 *Assistant Professor of Biostatician, Babol University of Medicine Sciences, Mazandaran, Iran.*

**Keywords:** Dysphonia, Hoarseness, laryngeal video endoscopy (video laryngoscopy), laryngeal neoplasm.

## Abstract

**Introduction:**

Laryngeal dysfunction may be divided into three categories; organic, neurologic and functional disorders. Dysphonia and hoarseness are the most common symptoms and, in some cases, the only signs of laryngeal dysfunction. In differential diagnosis of any type of chronic hoarseness, a neoplastic process must be considered and, thus continuous light video laryngoscopy can provide important information on the presence of neoplastic lesions in order to prevent disease progression via early detection and action.

**Materials and Methods:**

This cross-sectional, descriptive-analytical study was carried out on 197 patients with voice disorders admitted to Ayatollah Rouhani Hospital for video laryngoscopy. Available sampling was used and the results achieved were analyzed using SPSS17 statistical software.

**Results:**

A total of 197 patients (mean age, 40.72 ±15.17 years) participated in this study, 56.9% of whom were male. From analysis of video laryngoscopy, organic dysphonia was found to be the most common cause of voice disorders, while functional and neurologic dysphonia were observed in 8.6% and 5.6% of patients, respectively. Vocal nodules and Reinke's edema were among the most common causes of organic dysphonia, with a frequency of 24.4% and 23.4%, respectively; while laryngeal carcinoma accounted for 2.5% of all diagnosed cases with organic causes.

**Conclusion:**

Since the presence of voice disorders for more than 3 weeks can be a sign of laryngeal dysfunction, early diagnosis using noninvasive methods such as video laryngoscopy and appropriate medical measures can help prevent the disease progression and eliminate the need for actions such as laryngectomy.

## Introduction

Voice disorders are among the most common speech and language disorders (1), afflicting approximately 6% of children under 14 years of age, and 3–9% of the adult population (2). Several factors are involved in the development of dysphonia and hoarseness; in one classification, they can be divided into organic, neurologic, and functional categories (3). 

Voice disorders caused by organic lesions are associated with problems resulting from structural changes of the vocalization system (3) and are classified under the headings of malformation, traumatic, inflammatory/ infectious, and neoplastic (tumoral) etiologies (4). Functional dysphonia, which is classified into aphonia or psychogenic dysphonia, hyperfunctional dysphonia and hypofunctional dysphonia, is the other class of dysphonia in which no significant organic changes affect the voice producing structure (3-4). In neurologic dysphonia, innervation and muscular control of the vocalization system, from respiration to voice production, present deficits that are probably caused by a lesion in the central or peripheral nervous system (3). 

Since dysphonia and hoarseness are visible as the early symptoms of many disorders, and an appropriate treatment of diseases requires accurate diagnosis of the underlying causes, the examination and evaluation of the pharynx and larynx seems to be essential. Observation of the larynx and pharynx is an important part of a thorough examination of the head and neck. Although the location of these structures often inhibits direct observation, simple techniques can be used under clinical conditions for their evaluation (5), among which continuous light video laryngoscopy is a valid noninvasive and anesthesia-free approach. In comparison with indirect laryngoscopy, this method is easier and of greater accuracy and can therefore be used for the diagnosis of a wide range of diseases, including acute and chronic as well as benign or malignant disorders (5).

Most epidemiologic studies in the field of dysphonia have addressed the prevalence of this condition within specific age or occupational groups and few have investigated the causes of this disorder. In 2009 a study by Mendes et al., out of 73 teachers, 52% were observed to have hoarseness compared with 31.5% in the control group (non-teachers). In this study, risk factors were found to include cold air, surrounding sounds, loudness of teachers’ voices, excessive talking, and smoking (6).

In a 2008 study by Lopez et al., 579 teachers were selected as cases and 326 were selected as the control group. Teachers were first asked to fill out a standard questionnaire. They then underwent a thorough laryngeal examination that included a general assessment of the ear, nose and throat (ENT) along with a video laryngoscopy. The prevalence of dysphonia was 57% among the teachers involved in teaching; high pressure on the vocal cords caused by loud talking was the most common cause (18%), followed by vocal nodules (14%) and dysphonia caused by hyperfunction (8%). Organic lesions were three times higher among women than among men; in contrast, the prevalence of chronic laryngitis was three times higher in men than women and a functional voice disorder was approximately two times higher in men (7).

According to a study by Silverman, 6–23% of school-age children suffered from hoarseness, and the presence of vocal nodules was reported in most cases (8).

Finally, in a study conducted in Al-Noor Hospital in Saudi Arabia during 2005–2006, 30 patients with complaints of hoarseness referred to ENT clinics were investigated; dysphonia was associated with hyperfunction in the larynx in 26.7% of patients, phonesthesia in 16.7% and vocal-cord polyps in 13.3% of participants (9).

## Materials and Methods

This cross-sectional, descriptive-analytical study was carried out on patients admitted to the ENT and Speech Therapy Clinics of Ayatollah Rouhani Hospital affiliated to Babol University of Medical Sciences from April 2010 to October 2012. No criteria were applied for patients’ exclusion from the statistical population. 

Patients with voice disorders were first examined by an ENT specialist and, after giving informed consent, demographic information was obtained following referral to the Speech Therapy Unit. Participants were then prepared for laryngeal video endoscopy (with lidocaine gel or spray if required), and finally a video was prepared across four stages including respiration, transition from respiration to sound production or voice onset time (VOT), phonation, and coughing. Finally, a diagnosis was made by an ENT specialist and a Speech Therapist. The diagnosis- associated data were analyzed using SPSS_17_ statistical software. 

## Results

In this study, 197 patients with a mean age of 40.72 ±15.17 years participated, among whom 112 were male and 85 were female. A total of 116, 38, and 65 patients reported a history of reflux, paraneoplastic neurologic disorder (PND), and voice abuse respectively. Cigarette smoking, hookah smoking, and alcohol consumption were observed in 59, 39, and 31 patients respectively; 42 patients had high-risk jobs (teacher, lawyer, bank clerk, for example).

The primary objective of the present study was to investigate the causes of voice disorders, and the results achieved demonstrated that, with a prevalence of 85.78%, organic dysphonia was the most common cause of voice disorders. 

Functional and neurologic dysphonia was observed in 8.6% and 5.6% of patients, respectively. The results are presented in (Table 1).

**Table 1:** The prevalence of video-laryngoscopy findings in patients with voice disorders

Dysfunction	Organic 169 (85.78%)													Functional 17(8.63%)		Neurologic 11 (5.63%)	
	Inflammatory 79 (46.75%)						Benign tumoral 81 (47.93%)				Malignant tumoral 5(2.96%)	Trauma 4(2.3%)		‒		‒	
	Reinke's edema	Contact ulcer	Chronic laryngitis (nonspecific)	Sulcus vocalis	Other infections (candidiasis)	Reflux laryngitis	Vocal nodule	Polyp	Cyst	Granuloma	Laryngeal Carcinoma	Granuloma caused by intubation	Other traumas	Hyperfunction	Hypofunction	Right vocal-cord paralysis	Left vocal-cord paralysis
Frequency	46	17	12	2	1	1	48	27	5	1	5	2	2	2	15	4	7
Percentage of subcategory	58.2	21.5	15.13	2.54	1.27	1.27	59.2	33.3	6.1	1.2	100	50	50	1	7.5	---	---
Percentage of category	27.2	10.6	7.1	1.1	0.6	0.6	28.4	15.9	3	0.6	2.9	1.1	1.1	11.7	88.3	36.4	63.6
Percentage of Total	23.4	8.6	6.1	1	0.5	0.5	24.4	13.7	2.5	0.5	2.5	1	1	1	7.6	2.03	3.6
	40.1						41.1					2		8.6			

Among the causes of organic dysphonia, tumoral and inflammatory lesions were the most and trauma was the least prevalent types. Among benign tumoral lesions, vocal nodules and granulomas were respectively found to have the highest and the lowest prevalence. In terms of malignant lesions, 2.5% of the population studied had laryngeal carcinoma, all of whom were men aged >60 years. Further, in the category of inflammatory lesions, Reinke's edema was the most prevalent. Contact ulcers and nonspecific laryngitis was found in 8.6% and 6.1% of patients, respectively. The lowest incidence of inflammatory lesions was associated with sulcus vocalis, reflux laryngitis, and other infections (candidiasis). Among the causes of functional dysphonia, hypofunctional dysphonia was the most common cause of voice disorders, while in terms of neurologic dysphonia, left vocal-cord paralysis was the most common underlying factor.

## Discussion

In the present study, the highest prevalence of voice disorders was observed among patients aged <45 years. This is consistent with studies reported by Abtahi et al. (10) and Lopez et al. (7). Likewise, in other studies by Wang et al. (11), Roy et al. (12), Smith et al. (13) and de Medeiro et al. (14) the mean age of participants with voice disorders in the female group was similar to that in the present research; however, the mean age in the male group was slightly higher than 45 years. In contrast, in a study by Dabirmoghadam, the mean age of men with voice disorders was found to be under 45 years, which is congruent with the present research (15).

In this study, the prevalence of voice disorders was higher in men than in women, which is in line with the results achieved by Abtahi et al. (10). In studies by Lopez et al, Miller et al. and Thibealt et al. no differences were found between men and women with voice disorders; however, in studies by Dabirmoghadam et al., Lee et al. Roy et al. Smith et al. and Russell et al. the prevalence of voice disorders was higher among women (7, 15-20) Regarding the video-laryngoscopy findings in the present research, the prevalence of organic dysphonia was significantly higher than functional and neurologic dysphonia; however, these findings are not consistent with studies by Lopez, Urruikotxea and Sala (7,21,22) in which a higher prevalence was associated with functional disorders. The measures used for laryngeal examination may be the reason behind such a difference since the use of continuous light video laryngoscopy is suitable for the detection of organic lesions rather than functional lesions, which are better diagnosed using strobe light stroboscopy (6).

The results obtained in terms of the prevalence of tumoral lesions as well as the inflammatory lesions and underlying organic dysphonia are in a good agreement with the results of the study by Abtahi et al. in Isfahan(10). In the present study, the highest incidence of tumoral lesions was associated with vocal nodules. Likewise, in the studies by Lopez and Urruikoetxea,(7,21) the prevalence of vocal nodules was higher than other types of organic disorders; however, the prevalence of this lesion in the Sala study (22) lower than that of the present research. According to the findings of the current study, laryngeal polyps accounted for the most common benign tumoral lesions after nodules, with a prevalence higher than that reported in the Lopez and Dabirmoghadam studies(7,15). In terms of the malignant lesions, 2.5% of participants were observed with laryngeal carcinoma, which was much lower than that in the Ghahramani et al. and Abtahi et al. studies(10,23). In terms of inflammatory lesions, Reinke's **edema** was the most prevalent and, consistent with the studies of Lopez et al. and Dabirmoghadam et al.(7,15), was the second most common cause of voice disorders among organic dysphonia. In terms of the other nonspecific inflammatory lesions such as laryngitis, the results of the present investigation are consistent with the Dabirmoghadam study (15); however, the prevalence of granuloma and contact ulcers was higher based on our findings as compared with the Dabirmoghadam study (15). Similar to a study by Colton we found prevalence of sulcus vocalis (inflammatory category) to be >1% (24); however, a higher percentage was reported in a study by Poel (25). In the present investigation, a higher prevalence of hypofunctional dysphonia was observed when compared with hyperfunctional dysphonia, while a contradictory finding was reported in the Lopez study (7). 

This contradiction may be due to the lack of strobe light administered during laryngeal examination in this study, as this type of glottic closure is detectable when strobe light is applied. 

Moreover, in terms of the incidence of vocal-cord paralysis, the results of this research are consistent with those of Abtahi and Dabirmoghadam (10,15) whereas, in the Lopez investigation, the prevalence of voice disorders caused by vocal-cord paralysis was found to be much lower than that of the present research. Moreover, vocal-cord paralysis was more frequent on the left side than on the right side in this research, which is in line with studies by Shingo-Takano et al. Hirose et al. and Kelchner et al. and can be due to the length of the left recurrent laryngeal nerve and its resulting high vulnerability (26-28).

## Conclusion

As previously noted, organic dysphonia was the most common cause of voice disorder in the present study and may be significantly associated with a large number of risk factors underlying the mentioned disorders. Given the fact that an appropriate treatment will need to be etiologic in order to achieve optimal results, investigation into the risk factors underlying voice disorder, which *per se* indicates the presence of the disease, is of high importance. In addition, regarding the impact of laryngeal and voice disorders on patients’ quality of life, especially those who use their voice professionally, investigation, treatment, and prevention of the these disorders seem to be essential. Thus, the significance of a number of tests such as video laryngoscopy is an important area of research.
